# Involving the Family in the Care and Treatment of Women with Postpartum Psychosis: Swedish Psychiatrists' Experiences

**DOI:** 10.1155/2013/897084

**Published:** 2013-01-29

**Authors:** I. Engqvist, K. Nilsson

**Affiliations:** ^1^School of Life Science, University of Skövde, P.O. Box 408, 541 28 Skövde, Sweden; ^2^Department of Psychiatry, Skaraborg Hospitals, 521 85 Falköping, Sweden; ^3^Institute of Health and Caring Sciences, Sahlgrenska Academy, University of Gothenburg, 405 30 Gothenburg, Sweden

## Abstract

The aim of the study was to describe Swedish psychiatrists' experiences of involving the family in the treatment of women with postpartum psychosis. A qualitative design was used, and semistructured qualitative research interviews were conducted with nine psychiatrists from the south of Sweden. Data were analysed using qualitative content analysis. Four categories were found: *the family as a resource*, *the family as coworkers*, *preparing the family for the future*, and *the family as a burden*. The result showed that the psychiatrists considered the family to be a resource to which they devoted a great deal of care and effort. It was particularly important to involve the partner, informing about the course of the illness and the steps that need to be taken in the event of a relapse and reducing any guilt feelings. The psychiatrists instilled confidence and hope for a future of health and further child bearing. The family members' limited understanding of the treatment may impede the involvement of the family. Conclusion of the study was that the goal for family involvement was to facilitate the women's care and treatment. Further studies are needed to provide suggestions on how to develop family involvement in the care of women suffering from postpartum psychosis.

## 1. Introduction

Suffering from postpartum psychosis (PPP) calls for all possible forms of support as this is a rare illness [1, 2]. Involving families in patient care and treatment has also been found to be important in the recovery of people with psychiatric disorders [3], not least PPP [4]. The involvement or participation of relatives in supporting the patient during the hospital stay is laid down in Swedish law [5]. According to law, involuntary psychiatric treatment should be provided as far as possible with the cooperation of the patient and his or her relatives [6]. The Swedish National Board of Health and Welfare [7] describes this participation as the patient's right to be given individually tailored information, a choice of treatment options, and the opportunity for a second opinion. The regulations of the National Board of Health and Welfare [8] state that the quality system must ensure that patients and their relatives are informed and are involved in the care process. In this study, the focus is on the experience of Swedish psychiatrists of involving family members in the care and treatment of women with PPP. A family member is defined as a close relative or next of kin. 

Family involvement has been investigated in relation to mental illness in general. Close family members maintain frequent contact with a person suffering from severe mental illness, such as a psychotic disorder, while the person's social network, such as friendships, often breaks down [9]. From the individual's perspective, the family's social and emotional support has been found to be of considerable value and an important factor in the recovery process [10]. In a review by Saunders [11], it was shown that family members could be an important resource. Psychiatric research shows that including the families in planning and care has a strong influence on mental health and matches or outweighs the risk factors. Even brief family involvement will improve health and recovery, reduce the risk of a relapse, and increase family wellbeing [3, 12]. 

As mentioned, the focus of this study is on psychiatrists' experience of involving the family when treating women with PPP. Even if this illness is quite rare—one-to-two women per 1,000 births develop PPP [13]—it is important to find how to care for and treat the women who are affected as this illness is severe. The consequences could be traumatic and sometimes result in suicide and infanticide [14]. The majority of cases occur within the first few weeks [15] after childbirth, with a rapid onset of delusions, hallucinations, and impaired reality [16, 17]. The prognosis for PPP is favourable. With treatment, the woman usually recovers within a few weeks, perhaps up to a year [13]. 

In Sweden, psychiatrists care for women with PPP in several stages in the care process. Women with PPP typically receive inpatient treatment at a hospital for a short period, followed by outpatient treatment [18–20]. In Sweden, the mother and baby are usually discharged from the postnatal unit 1–4 days postpartum. The majority of the stricken women are discovered before discharge, and the remainders are discovered by the child healthcare nurse or by relatives. In Sweden, there are no specialist mother and baby units, and it is normal for the mother and the newborn to be separated and for the baby to be cared for by the partner/husband or other close relative at home. Consequently, the child is not the focus of this study. Both inpatient and outpatient care is provided in collaboration with several other healthcare professionals, including nurses, psychologists, and social workers [21]. 

It has been found in some studies [22, 23] that the majority of family members have insufficient opportunity to be involved in the patient's treatment. In a study from USA [24] covering 453 clinicians, it was found that the clinicians' attitude was poor with regard to participation by relatives in the care process. There were many barriers, including the attitude that relatives lacked interest, relatives were also mentally ill, and relatives had unrealistic expectations about the patient's progress. 

Contrary to these studies, Lakeman [25] found that the majority of family members were satisfied with their participation in the treatment. No studies have been found describe psychiatrists' or physicians' experiences of involving the families of women with PPP during the period of care and treatment. The aim of this study therefore is to describe Swedish psychiatrists' experience of involving family members when caring for women suffering from postpartum psychosis. 

## 2. Methods

A secondary analysis was conducted of the data collected from an earlier study that described the treatment approach employed by Swedish psychiatrists caring for women with PPP [18]. This study revealed that the psychiatrists focused on protecting women with PPP from suicide and infanticide. Treatment focused on maintaining patient safety along with prompt medical treatment. The reason for this secondary analysis was that the psychiatrists' statements gave the impression that questions concerning cooperation with the patient's family could be answered. A qualitative design was chosen for the project in order to shed some light on the care of women with PPP [26–28]. 

## 3. Sampling

The study was conducted in the south of Sweden. The psychiatrists were selected by means of convenience sampling combined with snowball sampling [28]. The criterion for participation was that the psychiatrists should have at least five years' professional experience to ensure that they had treated women with PPP. We commenced the study by contacting a psychiatrist and asking for help to find doctors who might be interested. We received three names, and these three provided the names of other doctors, all of whom were contacted. We contacted 12 psychiatrists in all and nine agreed to participate. Three of them subsequently declined because of a heavy workload. All the psychiatrists were contacted, and interviews were conducted by the first author (IE). 

## 4. Participants 

Nine leading psychiatrists, five male and four female, all in senior positions, participated in the study. Training to be a medical doctor in Sweden takes seven years and three months, and to be a trained specialist, that is, a psychiatrist, requires five years of further training. All psychiatrists in the study were trained in Sweden and were active in different areas of the country. Three of them had a Ph.D., which is an advantage when holding a top management or research position. In Sweden, about 15% of doctors have Ph.Ds [29]. Consequently, the participants had received more scientific training than psychiatrists in general. Two of the psychiatrists ran a psychiatric research programme, and one had previously conducted research on women diagnosed with PPP. 

## 5. Data Collection

Data were collected from October 2007 to February 2008 using semistructured qualitative research interviews with psychiatrists. They were contacted by telephone, whereupon the study was described and participation was requested. An introductory letter, describing the purpose of the study, was sent to the participants. Before the interviews were conducted, the participants were once again told about the purpose of the study, and written, informed consent was obtained. The interview guide was structured according to the following themes: how to involve the husband and the family, consideration to the husband and the family, and pros and cons of involving the husband and the family. These themes were introduced by asking the psychiatrists to give their views on each of the themes. Short and direct questions were used to probe for accuracy, clarity, and further details when necessary. Analysis of the data did not begin until all the interviews had been conducted. Eight psychiatrists were interviewed at their offices, and one was interviewed at home. The interviews lasted 35–70 minutes. 

## 6. Data Analysis 

The data were analysed using qualitative content analysis; both the manifest content of a text (i.e., what the text says) and the latent content (i.e., what the text talks about) [30]. In accordance with Graneheim and Lundman's description of content analysis, each audiotaped interview was transcribed verbatim. The text was then read several times to obtain an overall impression. In the next step in the analysis, the units of meaning (manifest content) were extracted from the text and with the study objective constantly in mind. These were then decontextualised and shortened. Finally, the condensed text was abstracted and given a code. The data and codes were compared consistently to identify similarities and differences (latent level). This comparison process resulted in putting the codes with associated units of meaning and similar content together into subcategories to recontextualise data, whereupon the final four themes were formulated. 

The authors contributed in the data analysis with their specific perspective. The first author (IE) is a psychiatric nurse. This may be seen as a limitation and preunderstandings may be difficult to eliminate. Therefore, the first author consistently tried to be aware of the preconceptions. The second author (KN) had limited experience in psychiatry and therefore provided objectivity in the analytic process. 

## 7. Ethical Considerations

Under Swedish law [31], approval from an ethics committee was not required at the time of the interviews. However, under this act and the Helsinki Declaration, participants are required to give their informed consent. Consequently, ethical considerations, as decided by the Swedish Research Council [31], were followed and the participants were informed about the study objectives and the data collection method. They were informed that participation is voluntary, that the information would be treated in confidence, and that they could withdraw at any time. All participants gave their written consent. 

## 8. Results 

The results describe the psychiatrists' experience of involving the family when caring for and treating women with PPP. 

For the psychiatrists, the predominant goal for family involvement was to facilitate the women's care and treatment. 

## 9. The Family as a Resource

All the doctors interviewed stressed the importance of the family as a resource that should be utilised and benefitted from during the woman's hospitalisation. To this end, they used a little extra persuasion to get the family involved. They stated that involving the partner and close relatives is vital in facilitating the woman's care and her recovery, and they placed considerable emphasis on family participation. If the family participates and is familiar with the care during the woman's hospitalisation, the follow-up care will also be strengthened due to their knowledge of the illness and what to expect during the recovery phase. 

When the woman was admitted to a psychiatric department, the task of informing and involving the family began. The person addressed in particular was the husband/partner, who received special attention from the clinicians. The doctors said that they explained to the husband/partner that the woman was ill, and he should not worry if she said strange things, such as he is not the child's father. They explained this by saying “*She means nothing by this; this is simply the brain acting freely"* [psychiatrist 4]. The doctors stated that early efforts to involve the family during hospitalisation will provide more confidence and strengthen the family's sense of concern and their appreciation for the ongoing care. It may also have implications for the care provided after hospitalisation:

*In the way we work here, we consider family involvement extremely important and I put a great deal of emphasis on this [psychiatrist 6].*

One of the doctors described his way of involving the family and the husband/partner in this way:

*Something I discovered early on, and which is extremely important, is to pay particular attention to the husband. By talking to him and by giving him his own point of contact…//… I make sure he has his own time with me, so that I can explain what it is all about, and tell him that the illness will fade away… and he should not bother about what she says right now, such as you are not the father of the child and a lot of other stuff. She is just sick. Usually they are at a complete loss. Things happen so quickly and frequently they do not understand anything [psychiatrist 5]. *

Considerable effort was made to educate and inform the family, for whom the illness was incomprehensible. They had perhaps never heard of the illness the woman was suffering from, and it was the responsibility of the doctors to inform and help the family to understand the true nature of the illness. They explained what a psychosis is, that is, that the brain leaves normal reality behind and sees, hears, and says things that are not normal reality. The illness often produces a sense of guilt among those who are ill and among their relatives. They blame themselves for the woman's illness. This applied mostly to the husband. The doctors felt it was vital to reduce the family's guilt, thus increasing their ability to accept the illness and its symptoms. At an early phase in the illness, the doctors pointed out the good prognosis. Information of this nature reduced the family's feeling of blame and guilt:

*I like to control the flow of information. Many of them will go home and straight on to the Internet. They go home and Google and end up on less serious websites that are there only to confuse the patients. I want to try to control this flow of information and provide written information that will allow them to read and learn. I sit down with them and give them an understanding of what is involved. I describe what a psychosis is and try to reduce the guilt that usually goes with it [psychiatrist 4]. *

## 10. The Family as Comorkers

When the doctor and the nursing staff in the team caring for the woman also bothered about and cared for the husband and other family members, the family in turn felt accepted and became partners in the woman's care. They felt appreciated and useful, which made it easier for them to provide support and assistance in the care process. The woman received support and the family could remain with her on the ward and when on leave from hospital. Being involved in the care, the family made sure she took her medication and they supported her in her own daily care as well as caring for her baby. Being involved created security. When the family acted as a partner for the physicians and health professionals, it could help to shorten the hospital stay. If a therapeutic relationship has been established, it will be easier for the family to return to the hospital in the event of a relapse:

*Yes, she received exemplary support from the husband and her parents. She received very good support [psychiatrist 1]. *

*It's important that you get them on board. Then they can be a valuable resource, even later on [psychiatrist 5]. *

Working with the family, cooperation with the sick woman usually improved. The family became a link to the woman. Most of the time, cooperation with the woman was difficult, with problems obtaining her consent regarding care and the different care decisions. If the family agreed, they in turn helped the woman to become more willing to participate in her care and approved healthcare decisions.

*You need to bring in the relatives so they can learn from early signs of the illness and try to establish the best possible relationship with the patient. I think most of the time this is not too difficult. They usually like it when I'm involved. If I'm there during the tough part, it's quite easy to take care of them later on as an outpatient [psychiatrist 2].*

Good cooperation with the husband and the family was built up by the doctor providing information and knowledge and establishing a good reputation. In turn, the family participated in the care and shared their knowledge about the sick woman's background. It became two-way communication. An exchange of experience would benefit the woman throughout the time she was ill, both in hospital and during her continued treatment.

*The father was really good and realised that she was ill. We worked well together and she was admitted to hospital for a week, maybe ten days [psychiatrist 3].*

When cooperation worked well, the husband felt it was positive to be involved in the various care visits during the time in hospital and later.

*In cases such as this, the husband needs to participate. When visiting and so on. This is true for visits by the doctor, the nurse, counsellor and so on [psychiatrist 7]. *

If the family did not understand or accept care decisions, it was more difficult to implement care and treatment. If the woman was subject to a compulsory care order, it was the doctor's decisions that counted although the doctors always tried to obtain the woman's and her relatives' consent even when she was under compulsory care. The optimal situation was if the woman and her family agreed to or at least understood that the decisions were necessary as a basis for the care that was being provided. If it became necessary to use coercive measures, such as medication with the aid of restraint or force, it was vital that the husband/family understood the importance of this type of care and that they were prepared for it:

*We always put her on compulsory care. She must always be under such care. It is by far the best for everyone. This is something that is very deep-seated … That's my personal opinion and approach [psychiatrist 5]. *

## 11. Preparing the Family for the Future

Instilling hope, joy, and encouragement for the future was a fundamental task of the doctor when providing information to the husband/partner—to instil patience and tolerance regarding the present situation and to make him believe in future together. The doctor needed to make the partner realise that despite the ongoing illness the woman would eventually recover and in the future be well enough to take care of her child and her family. There was a need to remind him that she is the mother and the best person for their child, and even though it may seem hopeless when the illness was at its most severe, the doctors felt that this was necessary. Once the illness had been treated and the woman recovered, there would be an opportunity to have more children:

*Provide him [the husband] with information and encouragement. Get him on board and secure his consent to the treatment I know she needs. Instil faith and hope for their future [psychiatrist 5]. *

During the hospital stay, the doctors began providing information about how spouses/couples should approach a possible future pregnancy. They argue that it was necessary for different care professionals to work together although it was equally necessary for cooperation with the husband and family. They talked about the importance of the woman and her husband coming along for a visit prior to pregnancy to discuss support for the woman and how to build up a network of specialised prenatal care, including a psychiatrist, to support her. The husband/partner and other relatives around her needed to be involved in this networking:

*..//.. if you want to build something solid, you need a functioning network of contacts: prenatal care, us and the family [psychiatrist 9]. *

## 12. The Family as a Burden

The doctor did not always succeed in creating a useful and trusting relationship or affiliation with the relatives and the patient. Sometimes the families opposed the care that had been decided and failed to understand that the care was necessary for the woman to recover. At times, it was not easy to work with the husband and family. Sometimes it made the family determined to thwart the treatment. They complained and occasionally they even took the woman home to treat her there. Unless the women were under compulsory care, this could not be refused. Once in a while, the family acted wrongly and sometimes they did not understand any better. At times, the family was concerned for their loved one, and it could lead to incorrect behaviour. The family might make every effort to spoil the treatment and care.

*They were very aggressive and said: “She shouldn't be here; we'll take her home and we'll manage this.” The decision fell to me and I thought: “Fine, you do that!” Six hours later, they were back. They threw her into the ward and said: “Take care of her!” [psychiatrist 5].*

In the past, psychiatric care has been bad at including the family in the care process. Relatives were often left out and no one asked for their knowledge and input about their loved one. The psychiatrists stated that they had tried to alter this pattern, and they all wanted a good working relationship with the woman's husband and her family and their involvement in the ongoing care:

*The family is very important and those of us working in psychiatric care are incredibly poor at including them. We are very slack when it comes to making contact with relatives [psychiatrist 6]. *

## 13. Discussion 

The family plays an important role in the care of patients in all areas of healthcare, and this is true in psychiatric care. The aim of this study is to describe Swedish psychiatrists' experience of involving family members in the care of women with postpartum psychosis. Four categories were found: *the family as a resource; the family as co-workers; preparing the family for the future; the family as a burden.* These categories are discussed below.

In this study, all the psychiatrists interviewed were of the opinion that it is essential to involve the family in the care of a woman with PPP. Involving the family makes it possible to use their knowledge of the woman to facilitate her care and treatment. The husband/partner is particularly important and a great deal of effort is made to involve him and make him a partner in the ongoing treatment. This is in line with the findings of Sjöblom et al. [32] in a study of nurses working in an acute psychiatric setting. The study verified the importance of the family and noted that the family increased their understanding of inpatient care by providing information. This was also noted in the present study. Sjöblom et al. [32] is a study of nurses, but it is reasonable to assume that the same applies to doctors working in the same context. 

Providing information to or educating families of women with PPP is crucial to the recovery process. Families need educating in order to understand the course of the illness and to acquire coping and problem-solving skills. It also reduces the feeling of guilt that usually accompanies this illness, both for the women and their relatives. This is an urgent task for the doctors and is in accordance with earlier research, which suggested that information given and received is of considerable value in psychiatric care and can reduce the families' feelings of shame and guilt [32–34]. 

It is also noted in the present study that it is not always the case that the relatives of the patients have been seen and are included in psychiatric care. What are the implications of such attitudes for families in clinical practice? Stjernswärd and Östman [35] investigated the experiences of families living close to an individual with depression. They reported that families felt that they were not always considered or treated well by healthcare personnel. According to Wilkinson and McAndrew [36], families frequently felt excluded from acute psychiatric settings and sought a greater degree of participation in the care. In the present study, this was not noted among the doctors. They attempted to change this way of acting, aiming to establish a good working relationship with families and trying to increase the family's involvement in the ongoing care and treatment. 

In Sweden, it is normal for the mother and child to be separated when the mother is diagnosed with PPP. The illness is severe and joint care may be impossible for the first few days. It is generally the father who becomes the primary caregiver at home [18]. The bonding will have to wait for the mother's part, even if they may be able to meet again after a few days. The connection to the father becomes stronger and he may replace the absent mother entirely. According to Erlandsson et al. [37], fathers who cared for their infants considered the increased time with the child to be very valuable. Although longing and togetherness with the woman appeared in the spectrum, the father later experienced a stronger and more lasting bond with the child. As reported by Erlandsson et al. [37], the father-child relationship is deepened when the father assumes more responsibility while getting to know his child. If the mother is incapable of caring for the baby, scientific studies show the importance of the father taking over in order to interact and care for the child [38, 39]. 

All doctors are accustomed to working with husbands/partners and close relatives of the women. Since relative participation is enshrined in Swedish law [5], the doctors boost their efforts to involve families. The question is what do they really think? Do they find that using family members benefits care or do they emphasise family work so much because it is stipulated in law and they are compelled to use family members and involve and inform them? This is difficult to know and the interviews provide no real information. According to Blomqvist and Ziegert [33] in a study of nurses working in an acute psychiatric setting, it was found that the family was not always a priority in this caring context. Is there a difference between doctors and nurses? 

It is important to remember that the results are statements made by the doctors and are not what they might do or think in practice. In an interview, people say what they want to say at that moment [40]. The doctors clearly understand that the families can provide information and knowledge about the woman that they would not receive otherwise. It also came out in the interviews that relatives can be a nuisance, they do not always follow instructions, and they could have a detrimental effect on the care process. How do the doctors treat a husband who refuses to accept the treatment and does not grant his permission for the care that is being provided? According to this study, the doctors try hard to get on friendly terms with the husband and contact him at an early stage, inform him about the woman and her illness, and establish a good relationship with him. This is similar to a friendship, with the two of them pulling together and moving forward. If the doctor can have a trusting relationship with the husband, the husband could be of use during the hospitalisation period and later on in the follow-up care. If there is no desire from the family or the husband of cooperation and no approval of the woman's care, the decision how to act for the doctor depends on whether the woman is on compulsory care or on voluntary care. When on compulsory care the doctor's decision applies. If the woman is on voluntary care, she can leave the hospital and return home. If the illness worsens, she will return to the hospital on compulsory treatment. 

The doctors also recognise the need to provide hope and encouragement for the future by reminding the couple that the illness does have an end and a good prognosis. They also start preparing the couple early on for the next pregnancy by providing information. In a study by Ewertsson et al. [10], it is reported that the majority of relatives interviewed in the study stated that they had experienced a negative approach by the healthcare professionals, revealing a lack of confirmation and cooperation. This is not borne out in this present study. The doctors' statements, however, are in line with findings from an earlier interview study [41] of strategies employed by nurses in connection with women with PPP, which also identified the importance of instilling hope, encouragement and confidence. 

In this study, the limitation could be the small sample size (*n* = 9) although the interview data are rich, describing relevant experiences and providing a deep understanding of the phenomenon in question. The open-ended questions gave the psychiatrists an opportunity to reflect on their experiences and to speak freely. In addition, the interviewer's knowledge and experience in the field facilitated the interviews. The richness of the data came from openness and a mutual understanding of the research field. 

## 14. Conclusion

The conclusion of the study is that the goal for family involvement is to facilitate the women's care and treatment. The psychiatrists stated that they put a great deal of effort into involving the family in the woman's care and treatment and that they make a particular point of trying to include the husband in the care process with the aim of securing his approval and understanding. They tried to inform the family about the course of the illness and what steps to take if there is a relapse. They tried to instil hope and confidence in a prosperous future and further child bearing. The family members' limited understanding of the treatment may impede the involvement of the family. Further studies are needed to provide suggestions on how to develop family involvement in the care and treatment of women suffering from postpartum psychosis. 
